# Salvianolic acid-B improves fat graft survival by promoting proliferation and adipogenesis

**DOI:** 10.1186/s13287-021-02575-4

**Published:** 2021-09-17

**Authors:** Jia-Ming Sun, Chia-Kang Ho, Ya Gao, Chio-Hou Chong, Dan-Ning Zheng, Yi-Fan Zhang, Li Yu

**Affiliations:** Department of Plastic and Reconstructive Surgery, Shanghai Ninth People’s Hospital, Shanghai Jiao Tong University School of Medicine, No. 639, Zhi Zao Ju Road, Shanghai, People’s Republic of China 200011

**Keywords:** Adipose-derived stem cells, Salvianolic acid B, Fat graft survival, Adipogenesis, Proliferation

## Abstract

**Background:**

Our previous study proved that *Salvia miltiorrhiza* could enhance fat graft survival by promoting adipogenesis. However, the effect of salvianolic acid B (Sal-B), the most abundant and bioactive water-soluble compound in *Salvia miltiorrhiza*, on fat graft survival has not yet been investigated.

**Objective:**

This study aims to investigate whether salvianolic acid B could improve fat graft survival and promote preadipocyte differentiation. The underlying mechanism has also been studied.

**Methods:**

In vivo, 0.2 ml of Coleman fat was transplanted into nude mice with salvianolic acid B. The grafts were evaluated by HE and IF at 2 and 4 weeks posttransplantation and by micro-CT at 4 weeks posttransplantation. In vitro, the adipogenesis and proliferative activities of salvianolic acid B were analyzed in cultured human adipose-derived stem cells (h-ADSCs) and 3T3-L1 cells to detect the mechanism by which salvianolic acid B affects graft survival.

**Results:**

In vivo, the weights and volumes of the fat grafts in the Sal-B-treated groups were significantly higher than those of the fat grafts in the control group. In addition, higher fat integrity and more viable adipocytes were observed in the Sal-B-treated groups. In vitro, salvianolic acid B showed the ability to promote 3T3-L1 and h-ADSC proliferation and adipogenesis.

**Conclusions:**

Our in vitro experiments demonstrated that salvianolic acid B can promote the proliferation of adipose stem cells and enhance the differentiation of adipose stem cells. Simultaneously, in vivo experiments showed that salvianolic acid B can improve the survival rate of fat transplantation. Therefore, our research shed light on the potential therapeutic usage of salvianolic acid B in improving the survival rate of fat transplantation.

**Supplementary Information:**

The online version contains supplementary material available at 10.1186/s13287-021-02575-4.

## Background

Repair of congenital and acquired tissue defects is a difficult and important task in plastic surgery. The commonly used soft tissue filling materials in the clinic are autologous tissues and synthetic materials. The wide application of synthetic materials is limited due to poor organizational compatibility, obvious contour sense, nonpermanent implantation and rejection reactions [1]. Compared with synthetic materials, autologous tissue does not have these limitations.

Adipose tissue is one of the most important materials in autologous tissue [2]. Adipose tissue is a kind of autologous soft tissue repair material that holds many advantages, such as abundant sources, easy sampling, concealed scarring, simple operation, good histocompatibility and no foreign body reaction. Therefore, it is also one of the most commonly used tissue sources in plastic surgery [3]. Fat transplantation is widely used in plastic surgery of the breast [4], hip [5], facial filling [6] and scar treatment [7]. In 2019, The American Aesthetic Society reported that 102,155 autologous fat grafting procedures were performed for buttock augmentation (34,086), breast reconstruction (24,892) and face filling (43,177), demonstrating a steady upward trend from previous years [8]. However, autologous fat transplantation also presents inherent limitations, which means that the fat grafting has some disadvantages, such as high and unpredictable tissue absorption rates, which lead to uncertain efficacy. Meanwhile, autologous fat transplantation may involve a variety of complications, such as fibrous tissue formation, cyst formation, fat liquefaction necrosis and calcification, which may manifest after autologous fat transplantation [9]. Therefore, these disadvantages will influence the effect of autologous fat grafting.

With the development of fat grafting research, two key factors are involved in improving the survival rate of transplanted fat: accelerating the revascularization of transplanted adipose tissue (revascularization theory [10]) and promoting the differentiation of preadipocytes into adipocytes (preadipocyte theory [11, 12]). Revascularization theory holds that transplanted adipose tissue can only maintain its nutrient supply by infiltration of surrounding tissue fluid, which is only 150–200 microns away, until sufficient blood supply is established between the graft and the host tissue. However, the host's neovasculature usually grows into the graft 5 days after transplantation and can only invade the periphery of the graft. Therefore, the survival rate of transplanted fat can be improved to some extent by accelerating the reconstruction of blood supply in the transplanted body, increasing the blood supply of the transplanted body, shortening the ischemic period of transplanted adipocytes, and increasing the reconstruction of blood supply in the transplanted body. For example, adipose tissue-derived stromal cells (ADSCs) [13], stromal vascular fraction (SVF) [14, 15], VEGF [16], etc. are used to increase the blood supply of transplantation. However, there are some restraints, such as the need for in vitro culture, cumbersome operation, easy pollution, consumption of a large amount of fillable adipose tissue, and poor patient compliance. Nonetheless, the survival rate of large volume fat transplantation is not satisfactory even if these auxiliary methods are used.

From another point of view, preadipocyte theory suggests that compared with mature adipocytes, preadipocytes are smaller in size and more tolerant to ischemia and hypoxia. In the early stage of fat transplantation, many mature adipocytes are necrotic and apoptotic due to ischemia, hypoxia and malnutrition. Some mature adipocytes are transformed into preadipocytes. When the environment of the fat transplantation recipient area is improved and blood supply and nutrition are sufficient, preadipocytes differentiate into mature adipocytes again [12]. According to the theory of preadipocytes, we should promote the proliferation of poorly differentiated preadipocytes, transform them into mature adipocytes, and maintain the volume and weight of adipose tissue as much as possible to improve the survival rate of fat transplantation.

*Salvia miltiorrhiza* is an important traditional Chinese medicine that plays an important role in the treatment of cardiovascular diseases [17]. Our previous study found that *Salvia miltiorrhiza* can effectively improve the survival rate of free fat transplantation in a fat graft rabbit model [18], and the fat retention rate of patients with autologous fat transplantation was significantly improved (SM group vs. non-SM group, 60.06 ± 16.12% vs. 34.04 ± 11.15%) [19]. Second, *Salvia miltiorrhiza* can promote the differentiation of preadipocytes. Salvianolic acid B (Sal-B) is the most abundant and bioactive water-soluble compound in *Salvia miltiorrhiza*. Studies [20] have shown that salvianolic acid B increases the mRNA expression of adipogenic transcription factors, including PPARγ, C/EBPα and PPARα, in 3T3-L1 preadipocytes to increase glucose uptake and mitochondrial respiration, reduce glycerol release and promote adipocyte differentiation. However, the effect of Sal-B on graft fat survival and its specific mechanism have not been investigated. In this study, we attempted to investigate whether Sal-B improves the survival rate of fat transplantation by promoting preadipocyte differentiation.

## Materials and methods

### Human adipose-derived stem cell (h-ADSC) isolation

The use of human tissue was approved by the local ethics committee of Shanghai Ninth People’s Hospital, Shanghai Jiao Tong University School of Medicine, Shanghai, China. Adipose tissue particles were harvested during thigh liposuction from adult female human patients (*n* = 5; mean age, 28 years; mean BMI, 24.5) at the Department of Plastic and Reconstruction Surgery, Shanghai Ninth People’s Hospital. All tissues were waste materials collected as a byproduct of surgery, and the samples were immediately transported to the tissue culture laboratory for processing.

The collected fat particles were washed with phosphate-buffered saline (PBS) and allowed to stand for 5 min at room temperature, and then the lower layer of tumescent fluid and blood components was discarded. Then, the fat particles were mixed with an equal volume of prepared type IV collagenase solution (Gibco, USA). The solution’s final concentration was 0.2%, which was dissolved in low-sugar Dulbecco’s modified Eagle’s medium (low-sugar DMEM, HyClone, USA), and the solution was filtered twice with a 0.22 μm filter (Falcon, USA). Then, the samples were placed on a 37 °C shaker and shaken at a speed of 175 rpm for 1 h. After shaking, the solution was centrifuged at 1500–2000 rpm for 5 min and the supernatant and fat suspension were discarded. Ten milliliters of low-sugar DMEM with 10% fetal bovine serum (FBS, Gibco, USA) and 1% antibiotic–antimycotic (Gibco, USA) was added to the centrifuge tube (Falcon, USA), pipetted evenly and centrifuged at 1000 rpm for 5 min. The supernatant was discarded, and cell pellets were observed at the bottom of the tube. Three milliliters of FBS-containing medium was added and pipetted evenly, and then 8 ml of serum-containing medium was added, mixed well and inoculated in a 10 cm petri dish. The cells were then placed in an incubator containing 20% oxygen and 5% carbon dioxide at 37 °C.

### 3T3-L1 and h-ADSCs expansion

The culture medium was changed every 48 h, and when the cells reached approximately 90% confluence, the 3T3-L1 cells (ATCC, USA) and h-ADSCs were washed three times using PBS. These adherent cells were then passaged using a solution of 0.25% trypsin plus 0.02% ethylenediaminetetraacetic acid (EDTA) (Gibco, USA) for 1 min. The cell suspension was further divided into three culture flasks along with growth medium. Cells at passage three were used for the experiment.

### Adipogenic differentiation

The h-ADSCs from the 5 liposuction donors were mixed, and used for follow-up experimental research. 3T3-L1 cells and h-ADSCs at passage three were used for adipogenic differentiation, and the cell density was adjusted to 2 × 10^4^ cells/L. Once the cells reached confluency, the growth medium was switched to induction medium A. After three days, the medium was replaced by induction medium B (all from Cyagen Biosciences, USA). Induction A and B were mixed with various concentrations of salvianolic acid B (0, 10, 50, 100 μmol/L) (Sal-B, Selleck, USA) before use. The cells were used on days 4 and 8 for follow-up experimental research.

### Oil red O staining

To measure the degree of adipogenesis and differentiation, the cells were washed with PBS, fixed in 4% paraformaldehyde for 30 min at room temperature, and stained with fresh Oil Red O solution (Solarbio, China) for 30 min. Then, the stained cells were washed with PBS two times. Adipogenic differentiation was visualized by the presence of numerous intracellular lipid droplets using an inverted fluorescence microscope (Nikon, Japan). The image analysis was performed using ImageJ according to the method of Deutsch [21] and Lin [22]. After the scale of the image was established, the area of the lipid droplets was measured and was displayed by ImageJ as the surface area in square micrometers (μm^2^). Three fields were measured for each of the 3 experiments, and three technical replicates were performed in each of the 3 experiments. The significance of differences between the control and treated groups was set at *P* < 0.05 and assessed by ANOVA with GraphPad Prism 8 (GraphPad Software, La Jolla, CA, USA).

### Triglyceride assay

Lipid accumulation was used as a marker of adipogenic differentiation and was assessed through quantitation of triglycerides in the cell. Salvianolic acid B was added to the samples during differentiation. On days 4 and 8 of differentiation, the triglyceride level in the culture medium was measured by a triglyceride assay kit (Nanjing Jiancheng Bioengineering Institute, China) according to the manufacturer’s instructions. Briefly, the assay was initiated with the enzymatic hydrolysis of triglycerides by lipase to produce glycerol and free fatty acids. The glycerol released was subsequently measured by a microplate reader at a wavelength of 510 nm. The protein concentration was measured using the BCA concentration (Boster, China). The triglyceride content (mmol/gprot) = Triglyceride concentration (mmol/L)/Protein concentration (gprot/L).

### 5-Ethynyl-2′-deoxyuridine (EdU) proliferation assay

Cells were seeded in 24-well plates and incubated under standard conditions with various concentrations of salvianolic acid B (0, 10, 50, 100 μmol/L). Twenty-four hours after incubation, cell proliferation was detected using the EdU Cell Proliferation Assay Kit (Invitrogen, USA) according to the manufacturer’s protocol. Briefly, cells were incubated with 50 μM EdU for 2 h before fixation, permeabilization, and EdU staining. Then, cell nuclei were stained with Hoechst 33,342 (Invitrogen, USA) for 30 min. The proportion of cells that incorporated EdU was determined by inverted fluorescence microscopy (Nikon, Japan). The cells were counted manually in each field, three fields were counted for each of the 3 experiments, and three technical replicates were performed in each of the 3 experiments. The proportion of Edu + cells (%) = 100*The number of Edu + (Green) cells/The number of total cells(Hoechst33342 + cells, Blue). The significance of differences between the control and treated groups was set at *P* < 0.05 and assessed by ANOVA with GraphPad Prism 8 (GraphPad Software, La Jolla, CA, USA).

### Cell viability assays

The effects of salvianolic acid B on the viability of 3T3-L1 cells and h-ADSCs were tested by a Cell Counting Kit-8 (Beyotime, China) according to the manufacturer’s instructions. Briefly, 5000 cells/well were seeded in a 96-well plate. The cells were cultured in growth medium or adipogenic differentiation medium with various concentrations of salvianolic acid B (0, 0.1, 0.5, 1, 5, 10, 25, 50, 75, 100, 125 μmol/L). At 72 h of culture, 10% CCK-8 reagent was mixed with medium and added to each well. The 96-well plate was incubated at 37 °C for 2 h. The relative number of cells was measured by absorbance at 450 nm using a microplate reader (Thermo, USA).

### Flow cytometry

After 3 days in culture with various concentrations of salvianolic acid B pretreatment, h-ADSCs were resuspended in PBS buffer according to the number of cells (5000/ml).

For Annexin V and propidium iodide staining, 195 μL of cell suspension was mixed well with 5 μL Annexin V-FITC and incubated at room temperature for 10 min. The cells were washed with PBS and resuspended in 190 μL of deliquated binding buffer, and then 10 μL of 20 µg/ml propidium iodide was added. The samples were analyzed by flow cytometry using CytoFLEX LX (Beckman Coulter, USA). The data were analyzed by CytExpert (Beckman Coulter, USA).

### Nude mouse coleman fat graft model

The process of the animal experiment is shown in Additional file 2: Figure S2. All animal experiments were approved by Shanghai Ninth People’s Hospital, Shanghai Jiao Tong University, School of Medicine, Shanghai, China. Female nude mice (aged 6 to 8 weeks) were housed in individual cages with a 12-h light/dark cycle and provided with standard food and water ad libitum. The mice were randomly divided into three groups (6 mice per group): saline, 10 μmol/L, and 50 μmol/L. Each mouse was injected subcutaneously on the left and right flank of the back with 0.2 ml of Coleman fat using a 1 ml syringe with a blunt infiltration cannula. The grafts were injected into a spherical shape. The mice were locally injected with 0.2 ml of saline or salvianolic acid B (10 μmol/L, 50 μmol/L) once every 2 days. At 14 days, the grafts on the left back were harvested and carefully separated from surrounding tissue, and their volumes and weights were measured. The wounds were closed with size 6–0 nylon sutures, and antibiotic ointment was applied to the affected area for 1 week to prevent local infection. Each harvested sample was assessed histologically and immunohistochemically. At 28 days, the fat grafts were scanned via micro-CT, the grafts were harvested and carefully separated from surrounding tissue, and their volumes and weights were measured. Each harvested sample was assessed histologically and immunohistochemically.

### Histological analysis and immunofluorescence staining

Tissues were fixed in paraformaldehyde overnight, embedded in paraffin, and cut at a thickness of 5 μm and then stained with hematoxylin and eosin. We used the methods of Shoshani [23] and Yu [24] to evaluate the histologic parameters, such as cell integrity, tissue inflammation, presence of cysts/vacuoles, and the extent of fibrosis. Each parameter was scored as 0 = absence, 1 = minimal presence, 2 = minimal to moderate presence, 3 = moderate presence, 4 = moderate to extensive presence, and 5 = extensive presence. The scoring was performed independently by 3 authors who were unaware of the grouping.

For immunofluorescent staining, tissue sections were incubated with primary antibody against Perilipin (Proteintech, #15294-1-AP, China, 1:200) diluted in blocking solution overnight at 4 °C. After incubation with Alexa Fluor 488–conjugated goat anti-rabbit immunoglobulin G (Invitrogen, #A-21206, USA, 1:500), nuclei were stained with 4′,6-diamidino-2-phenylindole (Southern Biotech, USA). ImageJ software was used for quantitative analysis. The image analysis was refered to the website (https://imagej.net/imaging/image-intensity-processing) and the method of Keskin [25].

### RNA extraction and real-time RT-PCR

To investigate the adipogenic differentiation potential of 3T3-L1 cells and h-ADSCs, we assessed the transcriptional levels of PPARγ, C/EBPα and FABP4 in 3T3-L1 cells and h-ADSCs by real-time PCR assays. Initially, total RNA of 3T3-L1 cells and ADSCs was extracted using a total RNA miniprep kit (Axygen, USA), and RT-qPCR was performed with an ABI 7900HT system using SYBR Premix (Takara, Japan) according to the manufacturer’s instructions. mRNA quantification was performed using glyceraldehyde 3-phosphate dehydrogenase (GAPDH) for normalization. The SYBR green primers for qRT-PCR are listed in Additional file 4: Table S1.

### Western blotting

Cultured cells were lysed with RIPA buffer (Beyotime, China) supplemented with protease inhibitor (PMSF, Biosharp, China). Briefly, 20 μg protein was resolved by 10% or 12% SDS-PAGE and electroblotted in polyvinylidene difluoride membranes (Millipore Sigma, USA). The membranes were blocked with 5% nonfat milk at room temperature for 1 h. The separated proteins were then immunoblotted and probed with anti-glyceraldehyde 3-phosphate dehydrogenase antibody (Proteintech, China; #10494-1-AP, 1:5000), anti-CEBP alpha antibody (Abcam, UK; ab40764, 1:1000), anti-PPAR gamma antibody (Abcam, UK; ab59256, 1:1000), and anti–FABP4 antibody (Abcam, UK; ab92501, 1:1000) at 4 °C overnight. The next day, the membranes were incubated with peroxidase-conjugated secondary antibody (Abcam, UK; ab205718, 1:10,000) at room temperature for 1 h after washing with Tris-buffered saline with Tween 20 for 10 min three times. ImageJ software was used for quantitative analysis, which was conducted on immunoreactive bands. The number of experimental replicates and technical replicates are three times.

### Micro-CT analysis

The fat grafts were scanned via micro-CT (PerkinElmer, USA), and the fat grafts were analyzed by ProPlan CMF 3.0.

### RNA-Seq analysis

RNA sequencing samples were acquired after the addition of Sal-B (50 μmol/L) or solvent to h-ADSCs which were from two patients for 4 days and 8 days in differentiation medium. RNA quantity and quality were measured using a NanoDrop ND-1000. The cDNA library was constructed using KAPA Stranded RNA-Seq Library Preparation Kit (Illumina) following the manufacturer’s protocol. The final double-stranded cDNA samples were verified with an Agilent 2100 Bioanalyzer (Agilent Technologies, Santa Clara, CA). After cluster generation (TruSeq SR Cluster Kit v3-cBot-HS, Illumina), sequencing was performed on an Illumina HiSeq 4000 sequencing platform. Image analysis, base calling, and error estimation were performed using Illumina/Solexa Pipeline (Off-Line Base Caller software, version 1.8). Quality control was checked on the raw sequence data using FastQC (https://en.wikipedia.org/wiki/FASTQ_format). Raw data were preprocessed using Solexa CHASTITY and Cutadapt to remove adaptor sequences, ribosomal RNA, and other contaminants that may interfere with clustering and assembly. The trimmed reads were mapped to the corresponding reference genome using HISAT2 (version 2.0.4) for RNA sequencing, and StringTie (version 1.2.3) was used to reconstruct the transcriptome. Ballgown software was applied to calculate the fragments per kilobase of exon per million fragments mapped in RNA sequencing data and analyze differentially expressed genes, with the fragments per kilobase of exon per million fragments mapped ≥ 0.5 (Cuffquant) considered for the analysis. The cutoff for defining which genes were differentially expressed were foldchange greater than 1.5. The Gene Ontology functional and Kyoto Encyclopedia of Genes and Genomes pathway enrichment analysis was performed for differentially expressed genes using the Database for Annotation, Visualization and Integrated Discovery and Kyoto Encyclopedia of Genes and Genomes Orthology-Based Annotation System online tools (http://www.geneontology.org and http://www.genome.jp/kegg).

### Statistical analysis

In this study, all in vitro experiments were conducted 3 times. Single blinding was used for the statistical analysis. Two blinded data analysts independently analyzed the data. The final data were consistent between the two analysts. Data are expressed as the mean ± SD. The continuous variables between the two groups were compared by the independent samples t-test. One way ANOVA with the Tukeys post-hoc test was employed for pairwise comparisons among multiple groups. The significance of differences between the control and treated groups was set at *P* < 0.05 and assessed by GraphPad Prism 8 (GraphPad Software, La Jolla, CA, USA).

## Results

### Appropriate concentration of salvianolic acid B promotes the proliferation of 3T3-L1 cells and h-ADSCs

**Fig. 1:**
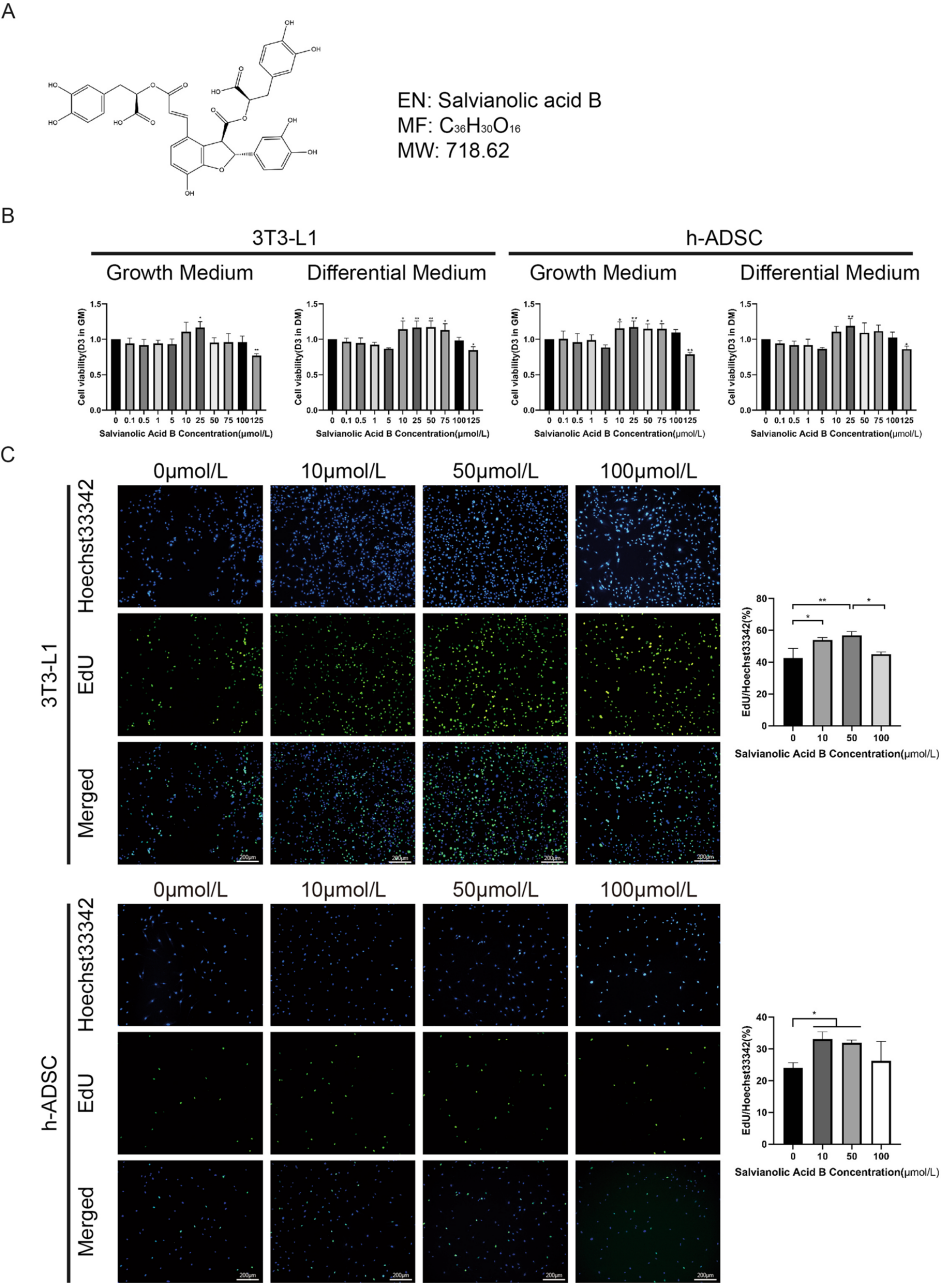
Effect of Sal-B on cell viability in 3T3-L1 cells and ADSCs. (**a**) Chemical structure of Salvianolic acid B (Sal-B). (**b**) Cell viability was determined with CCK-8 reagent after Sal-B treatment for 72 h (left: 3T3-L1, right: ADSCs). (**c**) EdU (green) proliferation assay was performed at 24 h after the addition of 10, 50 or 100 μmol/L Sal-B (PBS was added instead in the control group). Hoechst33342 (blue) staining of nuclei. Bar = 200 μm. The data represent the mean ± SD. * *P* < 0.05, ** *P* < 0.01. EdU, 5-ethynyl-2′-deoxyuridine; PBS, phosphate buffered saline; ADSCs, adipose tissue-derived stromal cells; SD, standard deviation

To explore the effect of salvianolic acid B on cell viability, 3T3-L1 cells and h-ADSCs were incubated in fresh medium containing different concentrations of salvianolic acid B. We observed that salvianolic acid B did not reduce cell viability with an appropriate salvianolic acid B concentration(less than 100 μmol/L) in growth medium and differentiation medium. However, when the drug concentration was as high as 125 μmol/L, the cell viability significantly decreased compared with that of the control group (3T3-L1 GM: 0.7706 ± 0.02934, ** *P* < 0.01; DM: 0.8469 ± 0.04764, * *P* < 0.05; h-ADSC GM: 0.7909 ± 0.02417, ** *P* < 0.01; DM: 0.8624 ± 0.04029, * *P* < 0.05) (Fig. 1b). Interestingly, we observed an increase in cell viability in the concentration range from 10 to 75 μmol/L, which might be related to the increase in cell proliferation.

Therefore, three different concentrations, 10, 50, and 100 μmol/L, were subsequently applied. To further study the effect of salvianolic acid B on cell proliferation, we incubated 3T3-L1 and h-ADSCs in different concentrations of salvianolic acid B. The results of EdU staining showed that the appropriate concentration of salvianolic acid B (10, 50 μmol/L) increased the proportion of EdU-positive cells in 3T3-L1 and h-ADSCs (3T3-L1: 10 μmol/L vs 0 μmol/L: 53.84 ± 1.626% vs 42.45 ± 6.093%; *P < 0.05; 50 μmol/L vs 0 μmol/L: 56.75 ± 2.440% vs 42.45 ± 6.093%, **P < 0.01) (h-ADSCs: 10 μmol/L vs 0 μmol/L: 33.06 ± 2.373% vs 23.97 ± 1.674%, *P < 0.05; 50 μmol/L vs 0 μmol/L: 31.85 ± 0.9125% vs 23.97 ± 1.674%, *P < 0.05) (Fig. 1c). This result indicates that salvianolic acid B can promote the proliferation of 3T3-L1 cells and h-ADSCs. In addition, through the flow cytometry apoptosis experiment (Additional file 1: Figure S1), we found that salvianolic acid B did not increase the apoptosis of h-ADSCs but might increase the apoptosis of 3T3-L1 cells when the concentration reached 100 μmol/L. In general, the appropriate concentration of salvianolic acid B (50 μmol/L) can increase the proliferation activity of adipose stem cells.

### Salvianolic acid B accelerates the adipogenic differentiation of adipose-derived stem cells

**Fig. 2:**
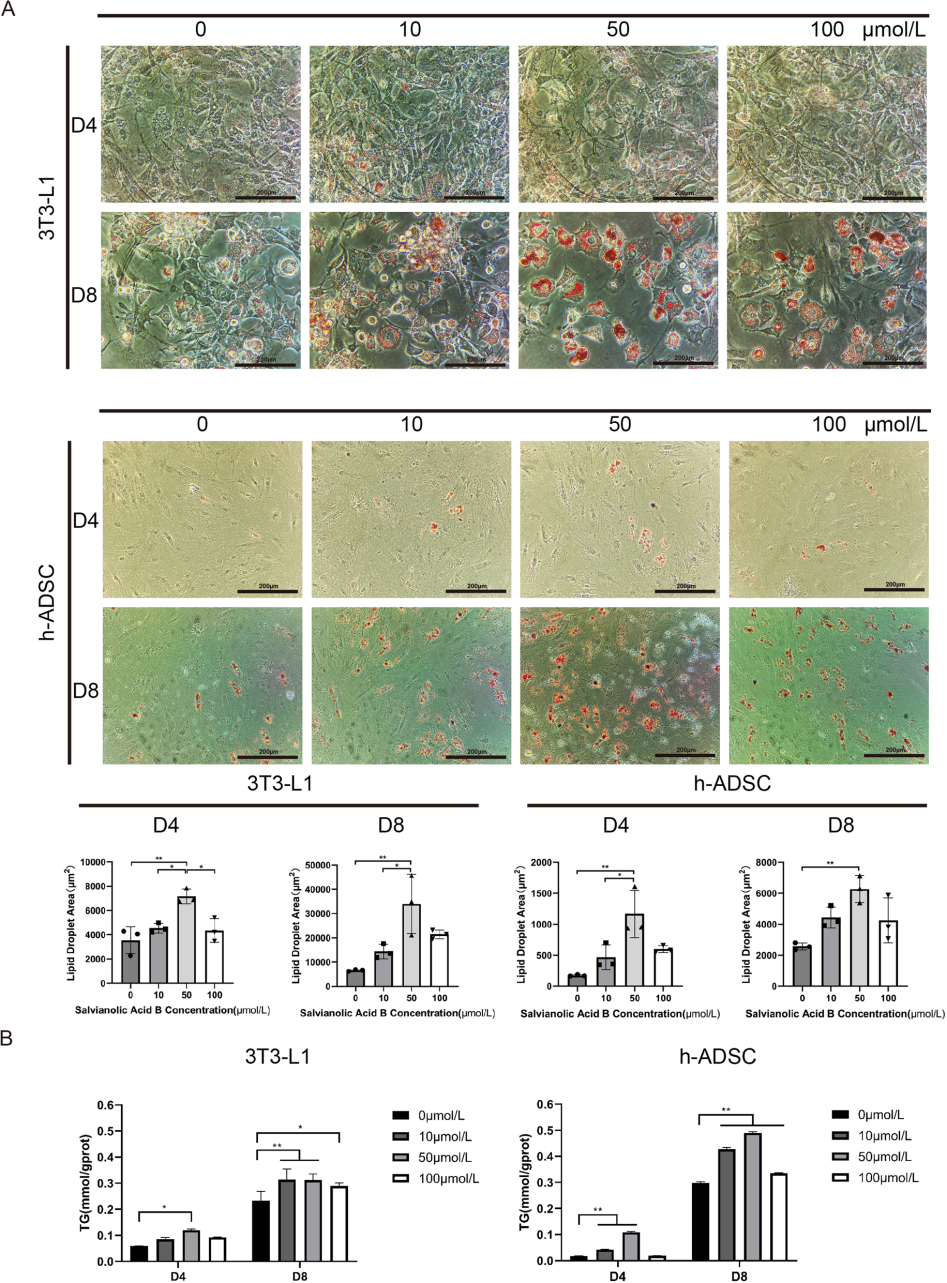
Effects of Sal-B on adipogenic differentiation in 3T3-L1 preadipocytes and ADSCs. (**a**) Oil Red O staining of various concentrations of Sal-B-treated 3T3-L1 adipocytes and ADSCs on day 4 and day 8. Bar = 200 μm. The area of the lipid droplets was measured and was displayed as the surface area in square micrometers (μm^2^). (**b**) Triglyceride content of various concentrations of Sal-B-treated 3T3-L1 adipocytes and ADSCs on day 4 and day 8. The data represent the mean ± SD. * *P* < 0.05, ** *P* < 0.01. ADSCs, adipose tissue-derived stromal cells; SD, standard deviation

On the 4th and 8th days of adipogenic differentiation of 3T3-L1 cells and h-ADSCs, the cells were stained with Oil Red O after cell fixation, and the triglyceride concentration was determined by a triglyceride kit. We found that different concentrations of salvianolic acid B promoted adipogenic differentiation. The effect of salvianolic acid B of 50 μmol/L in promoting adipogenic differentiation was the most obvious (Fig. 2a), and the determination of triglyceride content also confirmed this (D4 of 3T3-L1: 0 vs 10 vs 50 vs 100 μmol/L: 0.0588 ± 0.0006 vs 0.0847 ± 0.0074 vs 0.1197 ± 0.0044 *vs* 0.0925 ± 0.0006 mmol/gprot, *P < 0.05) (D8 of 3T3-L1: 0 vs 10 vs 50 vs 100 μmol/L: 0.2330 ± 0.0364 vs 0.3135 ± 0.0407 vs 0.3119 ± 0.0241 vs 0.2893 ± 0.0118 mmol/gprot, *P < 0.05, **P < 0.01) (D4 of h-ADSC: 0 vs 10 vs 50 vs 100 μmol/L: 0.0185 ± 0.0003 vs 0.0429 ± 0.0011 vs 0.1088 ± 0.0036 vs 0.0188 ± 0.0006 mmol/gprot, **P < 0.01) (D8 of h-ADSC: 0 vs 10 vs 50 vs 100 μmol/L: 0.2983 ± 0.0040 vs 0.4272 ± 0.0064 vs 0.4898 ± 0.0052 vs 0.3350 ± 0.0022 mmol/gprot, **P < 0.01) (Fig. 2b).

### Salvianolic acid B facilitates the survival rate of transplanted fat.

**Fig. 3:**
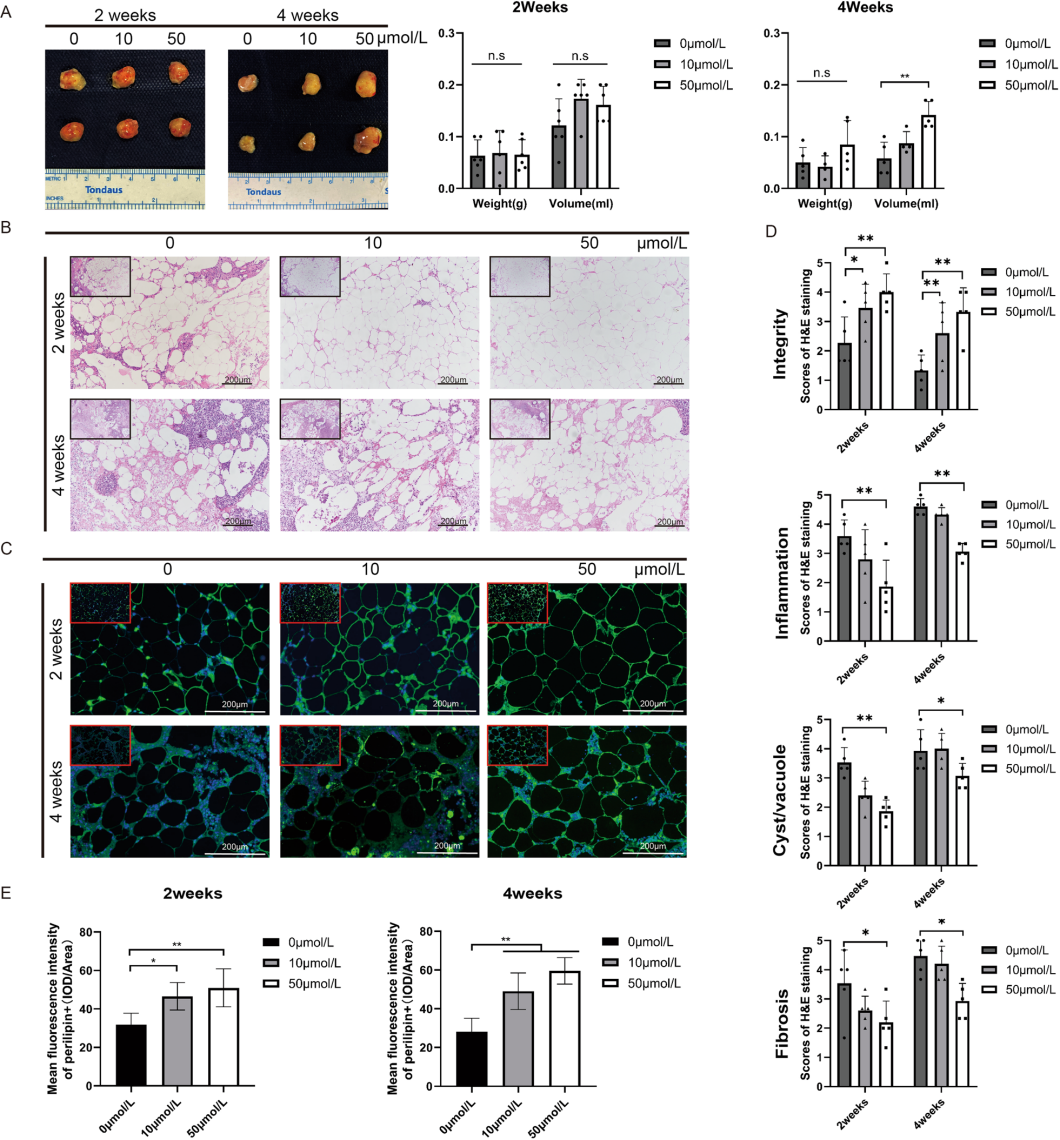
Sal-B promotes the survival rate of fat transplantation in a nude mouse Coleman fat graft model. (**a**) Macroscopic views of harvested tissue in the control and Sal-B-treated groups, which indicated the relative size of fat grafts at different time points (n = 5 per time point). (**b**) Images of H&E-stained tissue sections from the control group and Sal-B-treated group at different time points (scale bar = 200 μm). (**c**) Restoration of subcutaneous fat tissue in the Sal-B-treated group was confirmed by immunofluorescence staining of perilipin (green). DAPI (blue) staining of nuclei (scale bar = 200 μm). (**d**) Quantification of H&E Staining: cell integrity, tissue inflammation, presence of cysts/vacuoles, and the extent of fibrosis. (**e**) Quantification of immunofluorescence staining of perilipin, the level of the living adipocytes was measured by mean fluorescence intensity of perilipin (IOD/area). The data represent the mean ± SD. * *P* < 0.05, ** *P* < 0.01. SD, standard deviation

**Fig. 4:**
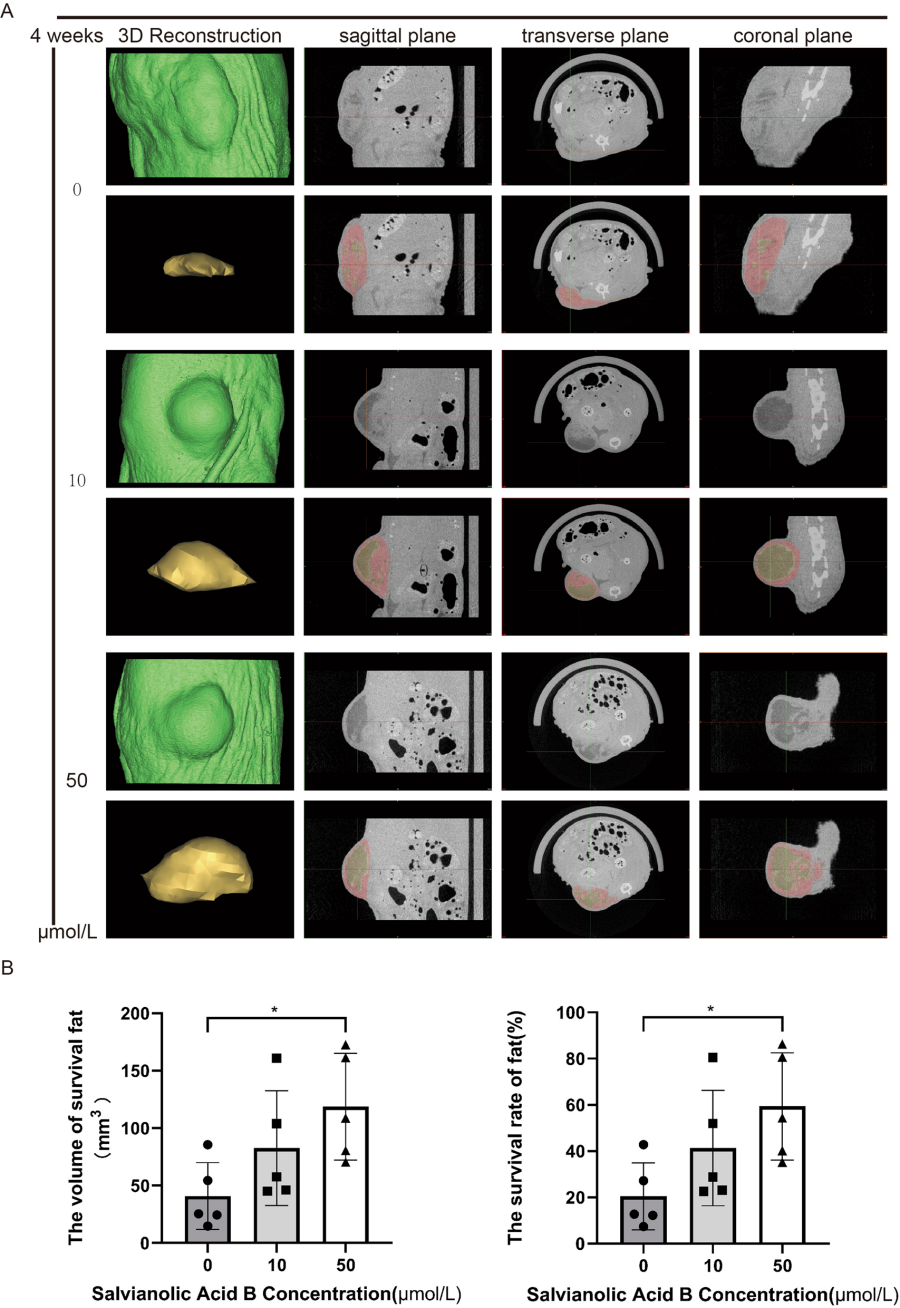
Analysis of fat transplantation in a nude mouse Coleman fat graft model (4 weeks) by micro-CT. (**a**) Micro-CT analysis of subcutaneous transplantation in three sections (sagittal section, transverse section, coronal section), where yellow represents surviving fat. (**b**) Measurement of the volume of surviving fat and survival rate by ProPlan CMF 3.0. The data represent the mean ± SD. * *P* < 0.05, ** *P* < 0.01. Micro-CT: microcomputed tomography program; SD, standard deviation

Our previous study found that *Salvia miltiorrhiza* could improve the survival rate of fat transplantation in fat graft rabbit models [18] and in patients with autologous fat grafting to the breast [19]. To explore whether salvianolic acid B, one of the main active ingredients of *Salvia miltiorrhiza*, plays an important role in improving the survival rate of fat transplantation, a nude mouse fat transplantation model was established. The nude mice were randomly divided into three groups: a control group (physiological saline) and two experimental groups (salvianolic acid B: 10 and 50 μmol/L). The results showed that in the second week, there was no significant difference between the control group and the experimental group. We attributed it to inflammation-led tissue swelling that made the difference not obvious. However, in the fourth week, we found that the volume retention rate of the 50 μmol/L salvianolic acid B treatment group was significantly higher than that of the control group (50 μmol/L vs control: 0.142 ± 0.026 vs 0.058 ± 0.031 ml, **P < 0.01) (Fig. 3a). In addition, to further explore the structural changes of the fat grafts, we conducted H&E staining and immunofluorescence detection. We found that the inflammatory cell infiltration level of the salvianolic acid B treatment groups was less than that of the control group in the second or fourth week, and the integrity of fat cells was better than that of the control group (Fig. 3b, d). Further, through immunofluorescence, the results showed that the number of Perilipin + living adipocytes in the salvianolic acid B treatment groups was significantly higher than that in the control group (2 weeks, 50 μmol/L vs 10 μmol/L vs control: 50.98 ± 9.87 vs 46.57 ± 7.12 vs 31.78 ± 5.99, *P < 0.05, **P < 0.01; 4 weeks, 50 μmol/L vs 10 μmol/L vs control: 59.59 ± 6.80 vs 49.08 ± 9.42 vs 28.10 ± 7.00, **P < 0.01) (Fig. 3c, e). By using micro-CT to analyze subcutaneous fat grafts, we found that salvianolic acid B increased the survival rate of fat grafts (50 μmol/L vs 10 μmol/L vs control: 59.36 ± 23.20% vs 41.36 ± 24.96% vs 20.45 ± 14.51%, * *P* < 0.05) (Fig. 4a, b). In general, salvianolic acid B can promote the survival rate of fat grafts.

### RNA-Seq analysis suggests that salvianolic acid B can promote adipocyte differentiation

**Fig. 5:**
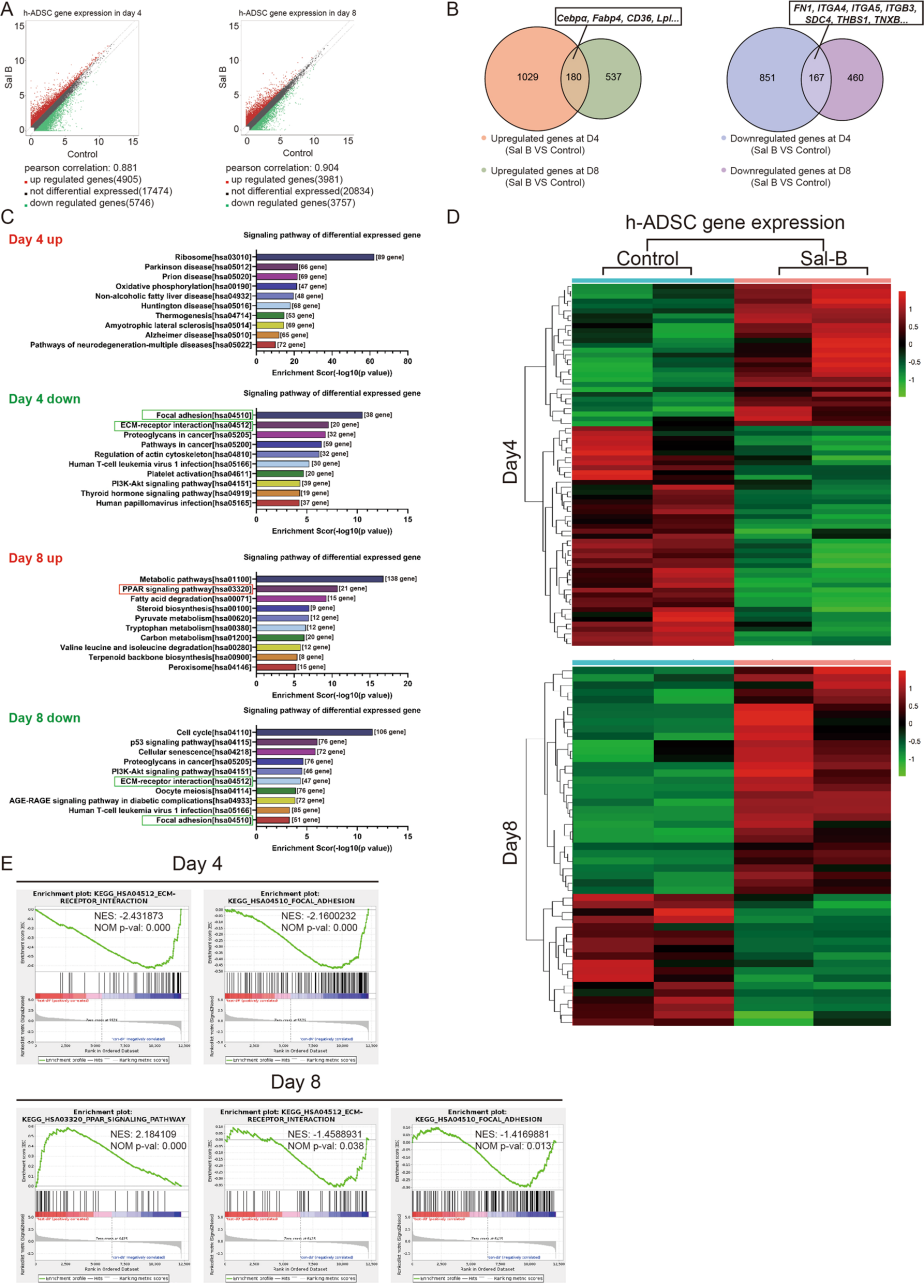
Transcriptomics data of ADSCs in the control group and Sal-B additional group. (**a**) Scatter diagram of differential expression analysis of RNA sequencing data from the control group and Sal-B additional group. The cutoff for defining which genes were differentially expressed were foldchange greater than 1.5. (**b**) Venn diagram depicting overlap between the 4th day and 8th day for both downregulated and upregulated genes. (**c**) KEGG analysis of differentially expressed genes on day 4 and day 8. (**d**) Heatmap of differential expression analysis of RNA sequencing data from the control group and Sal-B additional group in Day4 and Day8. (**e**) GSEA of the differentially expressed genes between the control group and Sal-B additional group on the 4th day and 8th day. KEGG, Kyoto Encyclopedia of Genes and Genomes; GSEA, Gene Set Enrichment Analysis

**Fig. 6:**
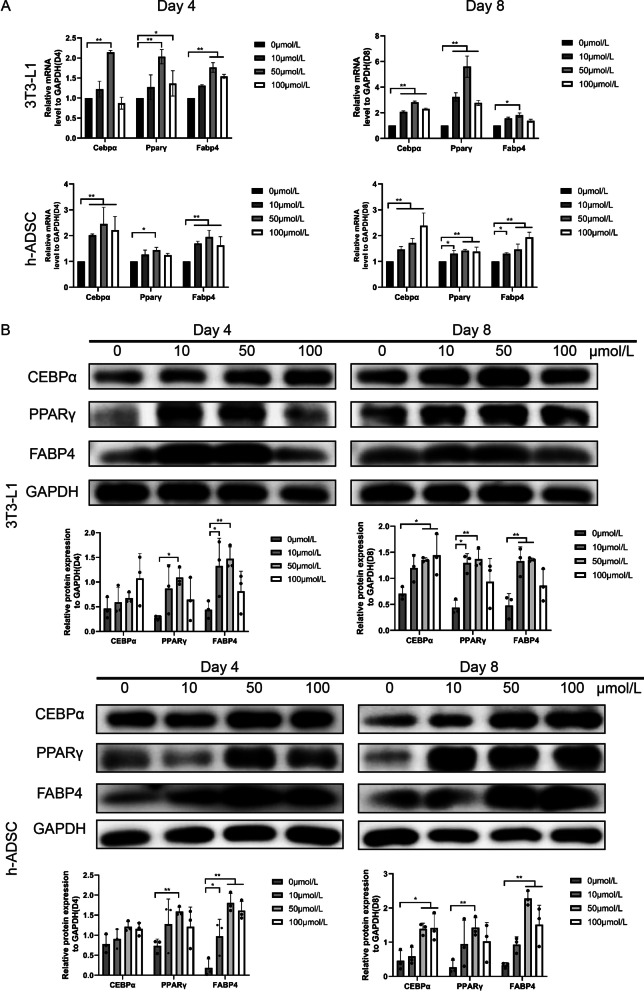
Effects of Sal-B on adipogenic differentiation in 3T3-L1 preadipocytes and ADSCs. (**a**) Relative mRNA expression of Pparγ, Cebpα, Adipoq and Fabp4 after Sal-B treatment in 3T3-L1 adipocytes and ADSCs on day 4 and day 8. (**b**) Relative protein expression of PPARγ, C/EBPα and FABP4 after Sal-B treatment in 3T3-L1 adipocytes and ADSCs on day 4 and day 8. The data represent the mean ± SD. *P < 0.05, **P < 0.01. ADSCs, adipose tissue-derived stromal cells; PPARγ: peroxisome proliferator activated receptor gamma; C/EBPα: CCAAT enhancer binding protein alpha; ADIPOQ: adiponectin; FABP4: fatty acid binding protein 4; SD, standard deviation

To further clarify the effect of salvianolic acid B on adipose stem cells, we collected cells on the 4th and 8th days and then performed RNA sequencing in both the case and control groups. The results indicated that large amounts of genes were differentially expressed between the case and control groups on the 4th and 8th days (Fig. 5a). On the one hand, adipogenesis genes, such as Cebpα, Fabp4, CD36, and LPL, were significantly upregulated (Fig. 5b). In addition, upregulated genes were enriched in the PPAR signaling pathway through KEGG analysis (Fig. 5c, Additional file 3: Figure S3A). On the other hand, studies have shown that extracellular matrix remodeling is necessary for adipose cell differentiation [26]. Notably, genes related to the extracellular matrix, such as FN1, ITGA4, ITGA5, ITGB3, SDC4, THBS1, and TNXB (Fig. 5b), were downregulated. Meanwhile, they were enriched in the ECM receptor response pathway through KEGG analysis (Fig. 5c, Additional file 3: Figure S3B). Furthermore, the focal adhesion pathway was also downregulated (Fig. 5c). It has been reported that reassembly of focal adhesion (FA) is essential for fat differentiation and that decreasing the expression of focal adhesion can improve adipogenesis [27]. Our experiments indicated that the expression of focal adhesion-related genes in the drug group was downregulated. Moreover, our DE heat map further confirmed that salvianolic acid B could change the expression of genes of pre-adipocytes in adipogenesis (Fig. 5d). At the same time, the GSEA results also verified the upregulation of the PPAR pathway and the downregulation of ECM- and FA-related pathways (Fig. 5e). In general, our sequencing results imply that salvianolic acid B is likely to promote adipose stem cell differentiation by enhancing the expression of adipogenic-related genes while restraining the gene expression of extracellular matrix-related pathways. The promoter effect of salvianolic acid B on the adipogenic-related genes described above was also demonstrated by quantitative RT-PCR and western blot (Fig. 6a, b). Meanwhile, at a concentration of 50 μmol/L, salvianolic acid B showed the best effect, which is consistent with our previous conclusion.

## Discussion

Autologous fat grafting has become a common technique for repairing volume and contour deficiencies in plastic and reconstructive surgery [28]. The biggest problem related to autologous fat transplantation is the unpredictable absorption rate [29]. In previous experimental studies, the absorption rate of fat transplantation was as high as 80% [30], which is consistent with the results of the control group in our experiment (survival rate in the control group: 20.45 ± 14.51%). Hence, it is still needed to further explore how to improve the survival rate of autologous fat transplantation.

In other studies, researchers have tried to improve the blood supply by using ADSCs [13], SVF [14, 15], and VEGF [16] to improve the survival rate of transplanted fat. However, there are some restraints, such as the need for in vitro culture, cumbersome operation, easy pollution, consumption of a large amount of fillable adipose tissue, and poor patient compliance. Nonetheless, the survival rate of large volume fat transplantation is not satisfactory, even if these auxiliary methods are used. In our previous research, we observed that a mixture of traditional Chinese medicine ingredients including *Salvia miltiorrhiza*, which contains Sal-B, can improve the survival rate of fat transplantation in a rabbit model and in patients with autologous fat grafting to the breast [18, 19]. In the present study, we identified Sal-B as the main active molecule in *Salvia miltiorrhiza* and showed that it can improve the survival rate of fat transplantation in a nude mouse fat graft model (50 μmol/L vs 10 μmol/L vs control: 59.36 ± 23.20% vs 41.36 ± 24.96% vs 20.45 ± 14.51%, *P < 0.05).

Researchers believe that later in fat transplantation, a large number of adipocytes, especially those near the center, become necrotic. Instead, preadipocytes proliferate and differentiate, producing new adipocytes to fill the necrotic area. Active cell proliferation appeared approximately 1 week after transplantation and lasted until 12 weeks after transplantation [12]. Therefore, researchers have suggested that enhancing the amount of preadipocytes in the graft or promoting the proliferation and adipogenic differentiation of more preadipocytes can retain more fat grafts, thereby improving the efficiency of transplantation. Our in *vitro* results demonstrated that Sal-B can promote the proliferation capability of 3T3-L1 cells and h-ADSCs and down-regulate the pathway of cell cycle/senescence and death pathways according to RNA-Sequencing. But high concentration such as 100 umol/L was actually preventing cell proliferation and showing more modest effects on adipose differentiation, since there was higher apoptosis in 3T3-L1 and lower TG production in both 3T3-L1 and h-ADSCs at D8. PPARγ and C/EBPα are key regulatory factors in adipogenesis [31]. PPARγ is necessary for inducing the differentiation of preadipocytes into adipocytes [32] and can promote adipogenesis in C/EBPα-deficient cells. In contrast, C/EBPα is unable to promote adipogenesis in PPARγ-deficient cells, which indicates that PPARγ is the main regulator of adipogenesis [33]. Otherwise, cross-regulation between C/EBPα and PPARγ is important for maintaining the differentiated state of cells [34]. In addition to PPARγ and C/EBPα, other transcription factors are also involved in adipocyte differentiation. These transcription factors function at different stages of adipogenesis to produce mature adipocytes. Among them, fatty acid binding protein 4 (FABP4) is one of the representatives, and it is responsible for the formation of mature adipocytes. In this study, through RNA-Seq analysis and subsequent PCR/Western blot verification, we revealed that Sal-B can promote the expression of adipogenesis-related genes, such as PPARγ, C/EBPa and FABP4, which indicated that Sal-B can promote ADSC adipogenic differentiation. Consistent with our findings, a recent study has shown that Sal-B can improve the mitochondrial activity of 3T3-L1 cells and increase the expression of adipogenesis-related genes [20]. This finding also revealed the potential of Sal-B in promoting adipogenesis. Finally we think the Sal-B could be injected into the local transplantation area or mix with fat graft in future clinical usages, such as breast reconstruction, face filling and other clinical usages.

In addition, we observed some interesting results in our experiment, and the degree of inflammatory cell infiltration in the salvianolic acid B-treated group was lower than that of the control group. Meanwhile, in the GSEA analyze of RNA-seq, we found the pathways of IL-17 and NF-kB were down-regulation in Sal-B treated group (Additional file 3: Figure S3C). Researchers have found that inflammation could inhibit adipogenesis [35]. Moreover, overactivation of inflammation reduces the survival rate of fat transplantation [12, 36, 37]. Therefore, salvianolic acid B may have an anti-inflammatory effect in fat transplantation, although this supposition requires more in-depth research. Otherwise, according to the results of RNA-Sequencing, we found that the salvianolic acid B could up-regulate the pathways of thermogenesis and oxidative phosphorylation in day 4 and metabolism genes in day 8, which was consistent with other researches in the field of the obesity studies [38, 39]. Therefore, we will conduct in-depth research on the anti-obesity mechanism of salvianolic acid B in subsequent studies.

## Conclusions

Salvianolic acid B can promote the proliferation of adipose-derived stem cells and enhance the differentiation of adipose stem cells by increasing the expression of adipogenesis-related genes. Our in vivo experiments indicated that salvianolic acid B can improve the survival rate of fat transplantation. Therefore, salvianolic acid B is a promising agent to improve the survival rate of fat transplantation.

## Supplementary Information

**Additional file 1**: **Figure S1**. Apoptosis was detected by a flow cytometer after treatment of ADSCs with Sal-B for 3 days. The data represent the mean ± SD. *P < 0.05, **P < 0.01.

**Additional file 2**: **Figure S2**. Nude mouse Coleman fat graft model. (**A**) Schematic diagram of the animal experimental design and schedule. (**B**) Mouse was injected subcutaneously into the left and right flanks of the back.

**Additional file 3**: **Figure S3**KEGG analysis of codifferential genes between day 4 and day 8. (**A**) Overlap between the 4th day and 8th day for both upregulated genes. (**B**) Overlap between the 4th day and 8th day for both downregulated genes. (**C**) GSEA of the differentially expressed genes between the control group and Sal-B additional group on the 4th day and 8th day.

**Additional file 4**: **Table S1**. Primers used for quantitative PCR.

## Data Availability

The datasets used and/or analyzed during the current study are available from the corresponding author on reasonable request.

## Abbreviations

BCA: Bicinchoninic acid
CEBPα: CCAAT enhancer binding protein alpha
CT: Computed tomography
DMEM: Dulbecco’s modified Eagle medium
ECM: Extracellular matrix
EdU: 5-Ethynyl-2′-deoxyuridine
FA: Focal adhesion
FABP4: Fatty acid binding protein 4
FBS: Fetal bovine serum
FITC: Fluorescein isothiocyanate
FN1: Fibronectin 1
GSEA: Gene set enrichment analysis
h-ADSC: Human adipose stem cells
HE: Hematoxylin-eosin staining
IF: Immunofluorescence
ITGA4, 5: Integrin alpha 4, 5
ITGB3: Integrin beta 3
KEGG: Kyoto encyclopedia of genes and genomes
PBS: Phosphate-buffered saline
PPARγ: Peroxisome proliferator activated receptor gamma
RNA: Ribonucleic acid
RNase: Ribonuclease
SDC4: Syndecan 4
SM: Salvia miltiorrhiza
SVF: Stromal vascular fraction
THBS1: Thrombospondin 1
TNXB: Tenascin XB
VEGF: Vascular endothelial growth factor
