# Supplementary figures and images for: Salvianolic acid-B improves fat graft survival by promoting proliferation and adipogenesis
Source: Stem Cell Res Ther. 2021 Sep 17;12:507. doi: 10.1186/s13287-021-02575-4 (PMC8447755; doi:10.1186/s13287-021-02575-4)

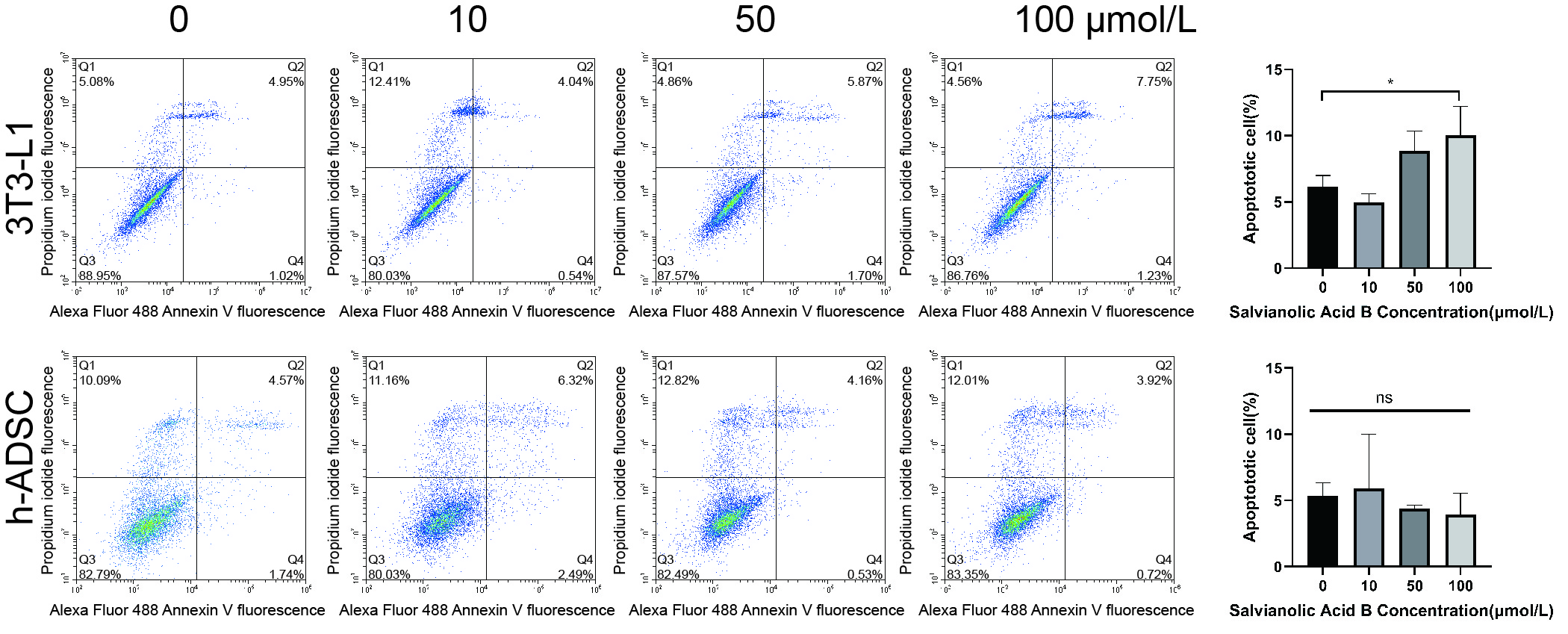

Supplement: Supplementary file 1 — Additional file 1: Figure S1. Apoptosis was detected by a flow cytometer after treatment of ADSCs with Sal-B for 3 days. The data represent the mean ± SD. *P < 0.05, **P < 0.01. [file 13287_2021_2575_MOESM1_ESM.jpg]

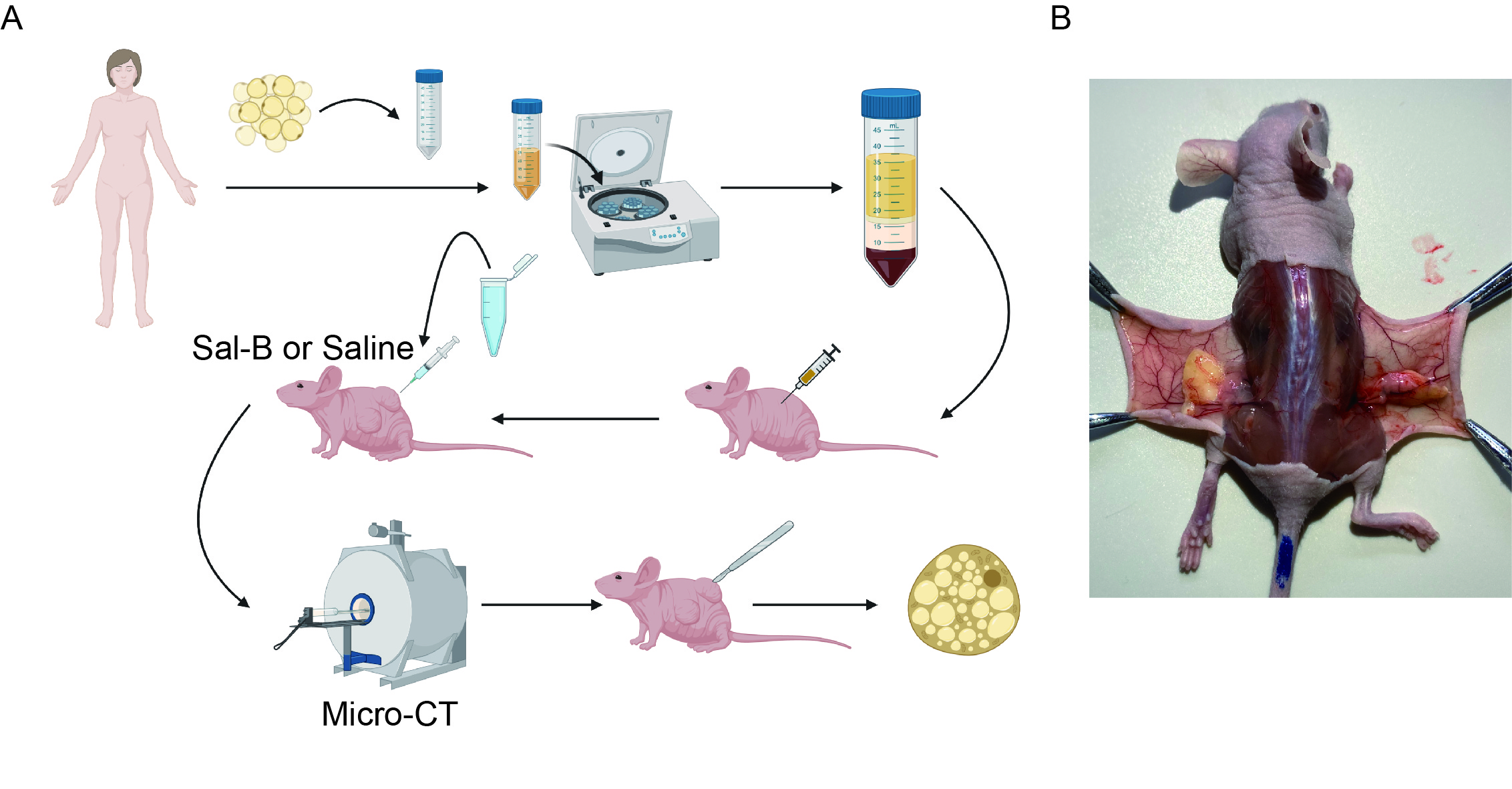

Supplement: Supplementary file 2 — Additional file 2: Figure S2. Nude mouse Coleman fat graft model. (A) Schematic diagram of the animal experimental design and schedule. (B) Mouse was injected subcutaneously into the left and right flanks of the back. [file 13287_2021_2575_MOESM2_ESM.jpg]

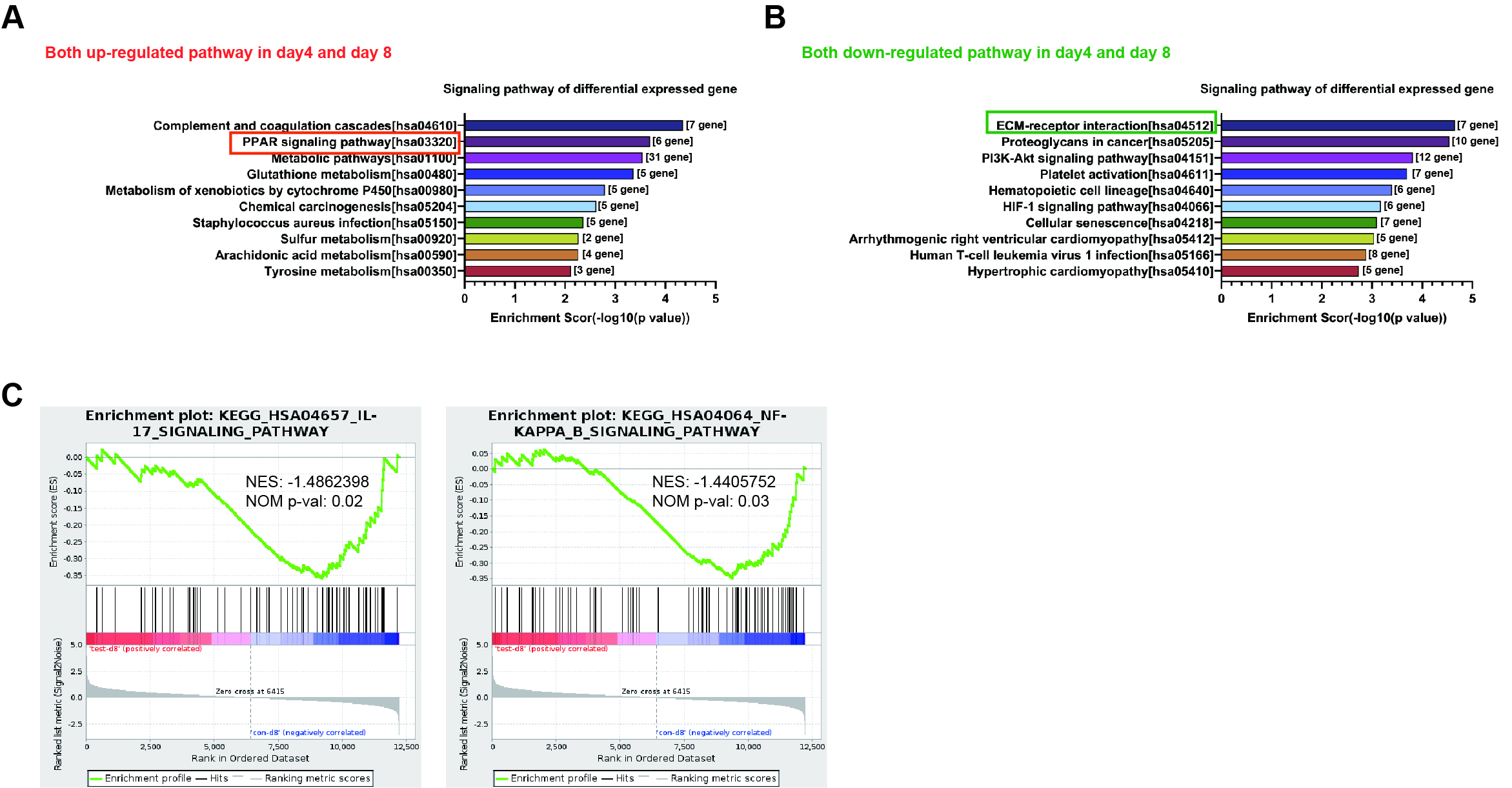

Supplement: Supplementary file 3 — Additional file 3: Figure S3KEGG analysis of codifferential genes between day 4 and day 8. (A) Overlap between the 4th day and 8th day for both upregulated genes. (B) Overlap between the 4th day and 8th day for both downregulated genes. (C) GSEA of the differentially expressed genes between the control group and Sal-B additional group on the 4th day and 8th day. [file 13287_2021_2575_MOESM3_ESM.jpg]
