# Supplementary material for: Salvianolic acid-B improves fat graft survival by promoting proliferation and adipogenesis
Source: Stem Cell Res Ther. 2021 Sep 17;12:507. doi: 10.1186/s13287-021-02575-4 (PMC8447755; doi:10.1186/s13287-021-02575-4)
Supplement: Supplementary file 4 — Additional file 4: Table S1. Primers used for quantitative PCR. [file 13287_2021_2575_MOESM4_ESM.docx]

| Gene | | House Mouse(5’-3’) | Human(5’-3’) |
| --- | --- | --- | --- |
| Pparγ | Forward | GGAAGACCACTCGCATTCCTT | GGGATCAGCTCCGTGGATCT |
|  | Reverse | GTAATCAGCAACCATTGGGTCA | TGCACTTTGGTACTCTTGAAGTT |
| Cebpα | Forward | GCGGGAACGCAACAACATC | TTGTATCTGGCCTCTGTGCC |
|  | Reverse | GTCACTGGTCAACTCCAGCAC | GCCGACGGAGAGTCTCATTT |
| Fabp4 | Forward | GAAGTGGGAGTGGGCTTTGC | ACTGGGCCAGGAATTTGACG |
|  | Reverse | TTGAGCTGAAGTCGCTTCCT | CTCGTGGAAGTGACGCCTT |
| Gapdh | Forward | CAGTGGCAAAGTGGAGATTGTTG | GGAGTCCACTGGCGTCTTCA |
|  | Reverse | TCGCTCCTGGAAGATGGTGAT | GTCATGAGTCCTTCCACGATACC |

Supplementary Table 1. Primers used for quantitative PCR
